# Editorial to “Utilizing the lid of SL sheath packaging for a water seal catheter insertion technique”

**DOI:** 10.1002/joa3.70035

**Published:** 2025-03-09

**Authors:** Yasushi Oginosawa

**Affiliations:** ^1^ The Second Department of Internal Medicine The University of Occupational and Environmental Health Kitakyushu Japan

Many studies have demonstrated the usefulness of catheter pulmonary vein isolation for atrial fibrillation (AF), and it has become a common and widely performed procedure. On the other hand, we still do not fully understand the pathogenesis of AF; therefore, we cannot guarantee that ablation will cure AF for a lifetime. Furthermore, AF itself is generally not an immediate life‐threatening or fatal emergency, and there are alternative treatments, such as drug therapy. Thus, catheter ablation for AF should be performed on a “safety‐first” basis.

AF ablation has a variety of complications, ranging from minor to fatal. Air embolism due to air withdrawal through introducer sheaths is a potentially serious complication.^1^ It is mainly caused by air entering the sheath during the insertion or replacement of a catheter under conditions of negative intrathoracic pressure. In fact, Tsukahara et al. experimentally verified that the amount of air drawn into a cryoballoon sheath during catheter insertion varies depending on the degree of negative pressure and the type of catheter being inserted, and we concluded that careful attention should be taken in situations of negative intrathoracic pressure and that the insertion of mapping catheters, especially those that are not recommended for use in cryoballoon sheaths, should be avoided.^2^

In Japan, AF ablation is rarely performed under general anesthesia with complete respiratory control. It is often performed under deep sedation with or without airway insertion, bi‐level positive airway pressure (BiPAP), or automatic servo ventilation (ASV). However, Ikoma et al. reported in a retrospective analysis of 381 patients who underwent respiratory management using deep‐sedation ASVs that negative left atrial pressure averaged −10.1 mmHg in 34.9% of patients.^3^ They concluded that negative left atrial pressure is not rare even with ASVs, so great caution should be exercised.

On the other hand, the “water seal” method, in which the sheath and catheter are submerged under water during the insertion of the catheter into the sheath, can theoretically completely prevent air retraction during sheath insertion regardless of left atrial pressure. A dedicated container for the water seal technique is already commercially available; however, this method is not widespread enough due to a lack of awareness, limited distribution, and its cost.

This time, Hayashi et al. reported a method of water sealing by using the shape of the Schwarz sheath package, which is commonly used for ablation, as a water bath.^4^ This method is feasible in all hospitals that commonly use Schwartz sheaths and should be considered to prevent unexpected air embolism, especially in patients suspected of having negative pressure in the left atrium due to respiratory issues.

Hippocrates once said, “First, do no harm” (*Primum non nocere*). This is a golden saying that should not be forgotten still in modern medicine. The water seal method is a primitive but extremely effective means of preventing air embolism, even if catheter ablation existed in the era of Hippocrates. Furthermore, it can be used in other procedures that require device insertion into a sheath under negative left atrial pressure. For example, in left atrial closure, which is currently performed by transesophageal echocardiography under positive pressure ventilation with a ventilator, deep‐sedation intracardiac echo‐guided implantation is already being attempted in other countries.^5^

Air embolism is a preventable complication. I hope that this article will raise awareness of air embolism as a potentially serious complication, including catheter ablation, and reaffirm the need for effective measures to prevent this serious complication.

## CONFLICT OF INTEREST STATEMENT

The author declare that there are no conflicts of interest.
